# Dietary Management of Skin Health: The Role of Genistein

**DOI:** 10.3390/nu9060622

**Published:** 2017-06-17

**Authors:** Natasha Irrera, Gabriele Pizzino, Rosario D’Anna, Mario Vaccaro, Vincenzo Arcoraci, Francesco Squadrito, Domenica Altavilla, Alessandra Bitto

**Affiliations:** 1 Department of Clinical and Experimental Medicine, University of Messina, Messina 98125, Italy; nirrera@unime.it (N.I.); cgpizzino@unime.it (G.P.); vaccaro@unime.it (M.V.); varcoraci@unime.it (V.A.); abitto@unime.it (A.B.); 2 Department of Human Pathology, University of Messina, Messina 98125, Italy; rdanna@unime.it; 3 Department of Biomedical Sciences, Dentistry and Morphological and Functional Images, University of Messina, Messina 98125, Italy; daltavilla@unime.it

**Keywords:** genistein, skin, estrogen receptor beta

## Abstract

In women, aging and declining estrogen levels are associated with several cutaneous changes, many of which can be reversed or improved by estrogen supplementation. Two estrogen receptors—α and β—have been cloned and found in various tissue types. Epidermal thinning, declining dermal collagen content, diminished skin moisture, decreased laxity, and impaired wound healing have been reported in postmenopausal women. Experimental and clinical studies in postmenopausal conditions indicate that estrogen deprivation is associated with dryness, atrophy, fine wrinkling, and poor wound healing. The isoflavone genistein binds to estrogen receptor β and has been reported to improve skin changes. This review article will focus on the effects of genistein on skin health.

## 1. Introduction

Aging is associated with a reduction in skin thickness and in the number of epithelial cells, with a concurrent decrease in stromal collagen [1]. The skin ageing process may intensify after menopause, and estrogen deficiency seems to have a direct effect on the epidermis. It seems that estrogen stimulation could encourage the proliferation of keratinocytes, leading to thickening of the epidermis, preventing its atrophy [2]. In the dermis, blood vessels would be stimulated again, and fibroblast production would also be stepped up, thus preserving the components that they secrete (e.g., collagen, elastic fibres, and glycosaminoglycans) [2,3]. Estrogen exerts its actions through two different receptors (estrogen receptors, ERs), also found in the skin. Both ERs are distinct proteins encoded by separate genes on different chromosomes. ERβ is located on human chromosome 14, whereas ERα is found on chromosome 6. Ligand binding activates the receptors that act as transcription factors by binding conserved estrogen response elements in the regulatory regions of target genes. Interestingly, many genes that are induced by estrogens lack estrogen response elements, including epidermal growth factor, epidermal growth factor receptor, and the cell cycle-associated cyclin D1. These genes belong to the group of secondary estrogen responsive genes, and estrogen affects their transcription/expression through activation of cytoplasmic signaling pathways such as Src/Shc/ERK. These secondary messengers are known to be activated by many transmembrane tyrosine kinase growth factor receptors, suggesting that estrogen effect may sometimes augment growth factor receptor activation [4]. In some cells, ERβ counteracts ERα, in some cases acting as an ERα heterodimer to inhibit the transactivating function of ERα, and in other cases acting as a homodimer to regulate specific genes, many of which are anti-proliferative [5]. The binding of estrogens to the ERs triggers specific responses in terms of proliferation activation and apoptosis arrest; nonetheless, some estrogen actions require the intervention of other hormones, such as progesterone and androgens. The variation in the distribution of receptors within the skin suggests that each has a different, cell-specific role. ERβ is the predominant estrogen receptor in adult human skin; it is strongly expressed in the stratum basale and stratum spinosum of the epidermis [6,7]. The same receptor is also found activated in fibroblasts from female skin, suggesting a strong involvement of ERβ in maintaining skin homeostasis [7]. Furthermore, estrogens prolong the anagen phase of scalp hair growth by increasing cell proliferation rates and postponing their transition to the telogen phase. ERα expression is limited to the dermal papilla cells of the hair follicle, whereas ERβ is found in the outer root sheath cells, epithelial matrix cells, dermal papilla cells, and the cells of the specialized bulge region of the outer root sheath [8]. It has been collectively demonstrated that ERα expression is quite low in the epidermis, while ERβ is abundantly represented.

Considering the presence and distribution of estrogen receptors within the skin and its annexes, it is not difficult to understand that hormone replacement therapy (HRT) can improve the menopause-related alterations. However, given the serious side effects of HRT (e.g., an increased risk of blood clots and certain types of cancer), a number of alternatives have been explored during the last decades, and the most intriguing seems to be the use of phytoestrogens. Isoflavones and lignans are the two main groups of phytoestrogens; their activity is generally weaker than endogenous estrogens, and they are not stored within the tissues [9]. Many phytoestrogens possess both agonist and antagonist estrogen properties, and their estrogenic activity is demonstrated through interaction with both ERα and ERβ [10]. Genistein (5,7,4′-trihydroxyisoflavone) is an isoflavone abundantly found in soy and other legumes, and acts as a selective estrogen receptor modulator (SERM), mainly binding to ERβ [11]. Genistein has a ~30-fold higher affinity for ERβ than ERα; when genistein is bound to ERβ, helix 12 does not adopt an agonist conformation, but instead has a position more similar to that seen with an antagonist. This result is unexpected because the molecular shape and volume of genistein and estradiol are very similar, and because genistein is a partial (60–70% of E2) agonist of ERβ. The conformation of helix 12 in the ERβ-genistein structure may account for the different effects exerted by genistein compared to estradiol.

Besides the specific estrogen receptor-related effects, genistein also acts as an antioxidant and as a specific inhibitor of tyrosine kinases, affecting many signaling pathways [12]. Genistein—as with all the other isoflavones—exists in two different forms: the glycosylated (genistin) and the aglycone (genistein). The aglycone genistein is absorbed from the intestine and conjugated with glucuronic acid during transport across the intestinal epithelial cells. After transport to the liver, the glucuronide may be excreted in the bile, whereafter it could re-enter the small intestine, allowing genistein to be deconjugated, absorbed, and metabolized for the second time [13]. In vivo bioavailability of genistein and its glycoside genistin indicates that genistein is readily bioavailable, while the glycoside derivative is poorly absorbed in the small intestine due to the higher molecular weight and hydrophilicity [14]. For this reason, the most studied form of genistein is the aglycone, which so far has been used in preclinical and clinical studies. Genistein supplementation in postmenopausal women is quite popular; in fact, several nutraceutical preparations have been launched in the market with very different amounts of genistein alone or combined with other isoflavones. However, the beneficial effects of genistein on menopausal features can be achieved at very specific dosage (54 mg/day), and only few preparations fulfill this requirement. In this review article are summarized the main effects of genistein on skin health from preclinical and clinical observations. This is a narrative review focusing on studies performed using genistein in vitro or in vivo to demonstrate an effect on skin. As search terms on Pubmed, we used “genistein and skin”, “genistein and fibroblast”, “genistein and wound healing”, and we selected all those articles with available full text. As a further criterion, articles using mixes of isoflavones without clear indications of genistein’s dose were not included.

## 2. In Vitro Evidence

### 2.1. Genistein Effects on Fibroblasts

Wound healing and scar formation are dynamic biological processes involving numerous cell–cell and cell–matrix interactions in a complex milieu of both local and systemic influences. In abnormal hyper-proliferating fibroblasts from hypertrophic scars, an important role is played by altered growth factors expression, dysfunctional receptors, and tyrosine kinase signaling dysregulation [15]. Fibroblasts obtained from hypertrophic burn scars were cultured with genistein at different concentrations (25, 50, 100 μmol/L) to inhibit their growth and proliferation through the blockade of the RAS (Rat sarcoma), Raf, ERK, and p38 proteins [16]. Genistein inhibited cell proliferation and activity by affecting nuclear translocation of phosphorylated ERK molecules; ERK mainly regulates cell proliferation, growth, and differentiation, while p38 is mainly related to stress and inflammatory reactions. These pathways are interconnected, and genistein may exert its suppressing effects on cells’ proliferation by interfering with them [16].

Abnormal scarring can also result in keloid formation, characterized by an imbalanced extracellular matrix (ECM) synthesis and degradation, a CTGF (connective tissue growth factor)-dependent fibroblast proliferation, and resistance to apoptosis. Normal human dermal fibroblasts (NHDF, Adult) and keloid fibroblasts (KEL FIB) were tested with different concentrations of genistein; the keloid fibroblast culture revealed an increase of CTGF mRNA and an increase of CTGF protein expression compared to normal fibroblast, confirming the contribution of CTGF in keloid fibroblast pathology [17]. Genistein decreased mRNA and protein expression of CTGF in keloid fibroblast in a concentration-dependent manner. Moreover, genistein decreases TGFβ1, β2, and β3 gene expression in keloid fibroblast, but its potential application as an antifibrotic factor in keloids treatment requires further research. Genistein did not induce p53 and p21 expression, and therefore it seems that it does not induce apoptosis in monoculture of keloids fibroblast. However, it revealed a cytoprotective effect, stimulating BCL-2 gene expression [17]. In another study, normal human epidermal keratinocytes (NHEK), NHDF, and KEL FIB were tested to investigate genistein as a potential regulator of C-JUN, C-FOS, and FOS-B of AP-1 subunits expression. C-JUN and C-FOS expression was lower in keloid fibroblast compared to normal fibroblasts cultured in control conditions. The study demonstrated that genistein modulated C-JUN expression in dermal keratinocytes and fibroblasts in a dose-dependent manner. The expression of C-JUN was significantly higher in keratinocytes treated with genistein compared to control cells [18]. These data further corroborate the potential efficacy of genistein for treating keloid scars.

Collagen synthesis by fibroblast is also crucial for maintaining skin homeostasis and healing of wounds. Genistein effects on collagen biosynthesis and the signaling pathways involved in its regulation were investigated in human dermal fibroblasts [19] under the oxidative stress conditions evoked by *t*-BHP (*t*-butylhydroperoxide). Genistein exerts biphasic effects on collagen biosynthesis; at 1 μM, it counteracted collagen biosynthesis inhibition evoked by *t*-BHP in fibroblasts. At 10 μM, it exerted a significantly diminished protective effect on collagen biosynthesis, whereas at 100 μM, it potentiated the inhibitory action of *t*-BHP. The study suggested that at nutritionally attainable concentrations (1 μM), genistein protects human dermal fibroblasts from oxidative stress-induced collagen biosynthesis inhibition. The mechanism of the protective effect of genistein on collagen biosynthesis in *t*-BHP-treated fibroblasts may be due to the prevention of disturbances in the IGF-I receptor-mediated, ERK1/ERK2-associated signaling pathway evoked by the oxidant [19].

### 2.2. Genistein Affects Glycosaminoglycan Synthesis

Aside from the tyrosine kinase inhibitory effect of genistein, another modulating activity was identified on glycosaminoglycan (GAG) synthesis by blocking the tyrosine kinase activity of the epidermal growth factor receptor [20] in fibroblasts from patients with mucolipidosis II (a mucopolysaccharidosis caused by deficiencies in enzymes involved in degradation of GAG). In this experimental condition, genistein—despite causing a significant reduction in GAG synthesis rate—ensures the maintenance of sufficient amounts of GAG, which is necessary for the proper functioning of cells and tissues [21]. In another mucopolysaccharidosis, the Sanfilippo disease, genistein was tested and compared to other flavonoids to reduce GAG synthesis and accumulation in fibroblasts obtained by affected subjects [22]. An inhibition of GAG synthesis was found in the presence of all tested compounds, though the most pronounced impairment of production of GAGs was observed in the presence of kaempferol, daidzein, and genistein; thus, the authors concluded by suggesting that these flavonoids alone or in combination might be a safe treatment option for mucopolysaccharidosis [22].

### 2.3. Genistein Effects on ultraviolet (UV)-Protection

Genistein was also tested for protection against UV-light exposure and prevented the UV radiation-dependent expression of cyclooxygenase-2 (COX-2) in HaCaT cells cultures suppressing both the basal and stimulated expression of cyclooxygenase-2, which suggests that it exerts anti-inflammatory activity [23]. In UVB-irradiated BJ-5ta cells are human skin fibroblast cells immortalized with hTERT, and besides reducing COX-2 expression, genistein also induced Gadd45 gene expression, thereby activating the DNA repair system [24]. The protective effect of genistein against senescence-like characteristics was also tested on human dermal fibroblasts (HDFs) following repeated subcytotoxic exposures to UVB to cause senescence. Genistein reversed the senescence process in HDFs, acting as an antioxidant through the down-regulation of p66Shc protein that involves forkhead protein suppression [25]. These data further suggest that genistein could be a good candidate ingredient for protective agents against UV-induced photodamage.

The effects reported by these in vitro studies have been summarized in Table 1.

## 3. In Vivo Studies

### 3.1. Genistein Effect on Photodamage

Most of the in vivo studies carried out in rodents evaluated the effect of genistein alone or combined with other flavonoids in reducing the damages associated with UV-light exposure. In a long-term (8 weeks) dietary supplementation study with isoflavone-rich fermented soybean (0.2083 μg genistein/mg of soybean), hairless albino mice exposed to UVB demonstrated a markedly reduced skin inflammation [26]. Topically applied genistein was shown to reduce the incidence and multiplicity of skin tumors in the dimethylbenz(a)anthracene initiated and 12-*O*-tetradecanoyl phorbol-13-acetate promoted mouse models [27,28]. In the UVB light-induced complete carcinogenesis model, topical pretreatment of mice with 10 μmol genistein significantly reduced the formation of H_2_O_2_ and 8-hydroxy-2-deoxyguanosine [27,28]. Because these are the precursors for free radicals, their attenuation is a significant step for chemoprevention.

The possible anti-nitrosative effect of genistein (10 mg/kg or 15 mg/kg, i.p.) in the prevention of skin injury and in the modulation of cell proliferation were tested in Hairless HRS/J mice following 24 h of UVB irradiation [29]. The most effective dose (10 mg/kg) demonstrated a reduction in lipid peroxides and nitrotyrosine, accompanied by upregulation of both PCNA and Ki67, which indicated that prevention of nitrosative skin injury promoted cell proliferation and DNA repair [29]. In another study, genistein potently inhibited the UVB-induced skin carcinogenesis and photodamage in hairless mice. The possible mechanisms of the anticarcinogenic action hypothesized by the authors include scavenging of reactive oxygen species, blocking of oxidative and photodynamic damage to DNA, inhibition of tyrosine protein kinase, downregulation of EGF-receptor phosphorylation and MAPK activation, and suppression of oncoprotein expression [30].

### 3.2. Genistein Effect on Wound Healing

In wound healing models, genistein was tested in excisional and incisional wound models. ICR mice receiving a genistein-enriched diet (0.025% and 0.1% genistein) for 2 weeks before wounding demonstrated a faster wound repair, likely stimulated by a modulated production of reactive oxygen species (ROS), which in turn downregulated the activity of NF- κB and TNF-α within the first 72 h following wounding. In ovariectomized 10-week-old C57/Bl6 mice, full thickness skin incisional wounds were systemically treated with 17-estradiol (0.05 mg, 21-day, slow-release 17-estradiol pellet, subcutaneously implanted) or genistein (50 mg/kg/day). Genistein substantially accelerated wound repair, associated with a dampened inflammatory response. Unexpectedly, co-treatment with the ER antagonist ICI had little impact on the anti-inflammatory and healing promoting effects of genistein [31,32]. Thus, the authors speculated that genistein’s actions are only partially mediated via classical estrogen receptor-dependent signaling pathways.

In ovariectomized rats chronically (6 months) treated with genistein (1 and 10 mg/kg/day, subcutaneously), full thickness incisional wounds were made and analyzed after 7 and 14 days [33]. Genistein was able to counteract the delayed wound healing, improving extracellular matrix remodeling and turn-over in OVX rats. Moreover, genistein (1 mg/kg) was more effective than estradiol or raloxifene on all skin parameters tested at days 7 and 14 after wounding [33]. The same authors also evaluated skin aging in 9-month-old OVX rats chronically treated with genistein, raloxifene, and estradiol [34]. OVX rats showed a decrease in TGF-b1, VEGF, MMP-2, MMP-9, tissue inhibitor of metalloproteinase (TIMP)-1 and TIMP-2 compared with sham OVX rats. All the treatments significantly restored this depressed molecular profile, but genistein (1 mg/kg) also significantly increased collagen thickness and skin breaking strength [34].

Collectively (Table 2), these data suggest that dosages as low as 1 mg/kg could be beneficial for treating skin disorders during estrogen deprivation, while higher dosages (up to 50 mg/kg) are needed where a normal estrogenic milieu is present.

## 4. Human Studies

In addition to all of the aforementioned preclinical evidence, it was also reported that the use of soy isoflavones may induce proliferation of the epidermis and increase dermal collagen [35].

In a double-blind placebo-controlled trial, 26 women in their late 30s and early 40s were randomly assigned to receive either an oral intake of 40 mg soy isoflavone aglycones per day or placebo for 12 weeks. It was observed that the isoflavones improved fine wrinkles and malar skin elasticity at the end of the study period [36]. Based on this observation, a randomized, double-blind, estrogen-controlled trial evaluated in 36 postmenopausal women the effect of a topical gel applied on the face, containing either isoflavone (4% genistein) or estrogen for 24 weeks [37]. Facial skin biopsies were taken from the preauricular area before and after the 24-week gel treatment; the cutaneous effects of isoflavone gel at six months were limited to an increase in dermal thickness and in the number of blood vessels, but the increments were smaller than those in the estradiol group. Furthermore, no change in hormonal vaginal cytology at 3 and 6 months was observed in comparison to baseline, suggesting the absence of a significant systemic effect [37]. Genistein was also tested in UVB-induced erythema (sunburn) in the dorsal skin of six men with skin type II to skin type IV. Genistein 5 μmol/cm^2^ was topically applied 60 min before and 5 min after UVB irradiation. The skin was photographed and quantitated for erythema, and genistein effectively blocked UVB-induced skin burns, suggesting its use as UV-protective agent [30].

Genistein was also used in an open-label study, where a group of 19 children with different subtypes of mucopolysaccharidosis type III, Sanfilippo syndrome, and different degrees of disability received genistein supplementation (5 mg/kg/day) for 1 year [38]. Treatment with genistein produced improvement in skin texture and hair morphology, reduced CoQ10, but did not modify GAG urinary excretion or the severity of the underlying disease. The treatment was generally well-tolerated and no secondary effects were observed. This study is of great importance because it demonstrates that genistein can also be safely administered to young subjects [38].

## 5. The Problem of Delivery

Topical delivery should help overcome the discrepancies associated with the relative bioavailability of genistein after oral administration, which has been observed in certain studies [39,40], but not in others [41]. However, genistein use in creams and topical applications may be affected by a low permeability rate, as reported by some authors [42]. To overcome this problem, drug delivery systems based on lipid nanoparticles have been employed. The most widely studied lipid nanoparticles are solid lipid nanoparticles (SLN) and nanostructured lipid carriers (NLC). Both have a solid lipid matrix, but NLC are prepared with a mixture of solid lipid and oil, which can increase drug loading and stability [43]. An experimental study compared SLN and NLC nanoparticles combined with genistein for controlled release in skin, and increased penetration in deeper skin layers. The genistein–NLC formulation was the most effective in favoring the penetration in to the deeper skin layers, suggesting that NLC could be a promising nanocarrier for the topical delivery of genistein [44].

Another well-studied approach exploited the use of polymeric gels—in particular, polyethylene glycol 400 (PEG 400). The formulation was tested for skin permeation from a saturated solution of dry soy extract [45]. Genistein flux increased significantly when pure genistein was used as a suspension in PEG 400, and resulted in significant skin retention. To further investigate the transdermal delivery of genistein, studies were conducted using pH 6 and pH 10.8 buffers and soybean oil as vehicles [46]. Of these, the calibrated deposition of genistein into nude mice and pig skin for a saturated solution in pH 6 buffer was higher than the other vehicles. Pretreatment of skin for 2 h with either oleic acid or α-terpineol as penetration enhancers did not increase the permeation of genistein at pH 6. It was reported that topical delivery was promising for genistein use against photoaging and photodamage, providing a possible application of these gels as skin protection agents for UV-induced damage and chemoprevention of melanoma [47].

Another approach exploited to improve isoflavone delivery into the skin was tried with microemulsions. The effects accumulation as well as the solubility of genistein were tested on pig skin using a water/oil-type microemulsion, and a significant protection against UV-induced oxidative damage was observed [48]. Genistein inhibited lipid peroxidation in guinea pig dorsal skin, as well as UV-B-induced erythema formation.

## 6. Conclusions

**Figure 1:**
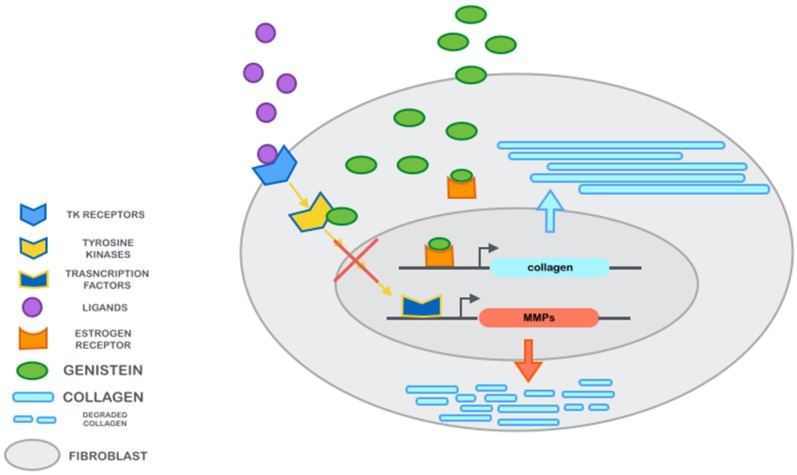
Mechanism of action of genistein in fibroblasts.

During menopause, as estrogen levels decrease, testosterone stimulates sebaceous glands to secrete thicker sebum, giving the appearance of oily skin; in addition, some women may develop facial hair, particularly in the chin area. As estrogen levels drop during menopause, fat deposits tend to redistribute over the abdomen and/or on the thighs and buttocks. The result is a loss of supportive fat below the skin of the face, neck, hands, and arms; this allows sagging wrinkles to appear, and the skin over these areas is less easily compressed as it loses its mobility. The lowered estrogen levels also result in less production and repair of collagen and elastin in the dermis (particularly if the skin is exposed to UV rays), resulting in elastosis. Estrogens also temper melanin production; their lack can result in brown “age spots” appearing on the face, hands, neck, arms, and chest of many women. All these changes lead to a faster aging of skin. Estrogen replacement prevents and ameliorates these features, but too many side effects have been related to their use. Over the past decades, genistein has been used as an alternative treatment for menopausal symptoms [49,50,51,52,53], and in light of the effects reported on skin aging (Figure 1), it could be considered as an effective alternative treatment for menopause. In conclusion, genistein might be a new potential therapy for the management of skin disorders as well as age- and menopause-related skin changes commonly observed in postmenopausal women. Considering the bioavailability following oral assumption and the permeability after topical application, it seems that oral administration of at least 50 mg/day of pure genistein without other isoflavones could be the best dosing strategy to obtain clinical efficacy.

## Figures and Tables

**Table 1:** Genistein effects on in vitro experimental models.

In Vitro Experimental Models	Genistein Effects
Fibroblasts from hypertrophic burn scars, normal human dermal fibroblasts (NHDF) keloid fibroblasts (KEL FIB), normal human epidermal keratinocytes (NHEK), NHDF stimulated with *t*-BHP (*t*-butylhydroperoxide) [16,17,18,19]	↓ RAS, RAF, ERK, p38
	↓ CTGF, TGFβ1, β2 and β3
	↑ BCL-2
	↑ C-JUN
Fibroblasts from patients with mucolipidosis II and Sanfilippo disease [21,22]	↓ GAG
HaCaT, BJ-5ta, and human dermal fibroblasts exposed to UV radiation [23,24,25]	↓ COX-2
	↑ Gadd45 gene expression
	↓ p66Shc protein

↑ and ↓ respectively indicate an increase or a reduction of the expression of the molecules. RAS, Rat Sarcoma; RAF, Rapidly Accelerated Fibrosarcoma; ERK, Extracellular signal-regulated Kinase; CTGF, Connective Tissue Growth Factor; TGFβ, Transforming Growth Factor beta, BCL-2, B cell leukemia 2.

**Table 2:** Genistein effects on in vivo experimental models.

In Vivo Model	Dose	Outcomes	Genistein Effects
UVB-irradiation and carcinogenesis models [26,27,29]	Dietary supplementation 0.2083 μg/mg of fermented soy beans 1, 5, 10, 20 μmol genistein topically applied Intraperitoneal injections 10–15 mg/kg	Anti-inflammatory effect in hairless mice with photodamage Reduction of skin tumorigenesis in mice Anti-nitrosative effect in hairless mice	↓ skin inflammation
			↓ H_2_O_2_
			↓ 8-hydroxy-2-deoxyguanosine
			↓ lipid peroxides
			↓ nitrotyrosine
			↓ tyrosine protein kinase
			↓ EGF-receptor phosphorylation
			↓ MAPK activation
			↓ oncoprotein expression
			↑ PCNA
			↑ Ki67
Wound healing models [31,32,33,34]	Dietary supplementation 0.25–1 g/kg Subcutaneous injections 50 mg/kg Subcutaneous injections 1–10 mg/kg Subcutaneous injections 1–10 mg/kg	Improving wound healing in intact mice Wound healing in OVX mice Wound healing in OVX rats Skin aging in OVX rats	↑ wound repair
			↓ ROS
			↓ NF-κB
			↓ TNF-α
			↑ TGF-β1
			↑ VEGF
			↑ MMP-2 and MMP-9
			↑ TIMP-1 and TIMP-2
			↑ collagen thickness
			↑ skin breaking strength
